# Draft Genome Sequence of Bacillus tropicus Strain UPM-CREST01, Isolated from the Bulk Paddy Soil at Kampung Gajah, Perak

**DOI:** 10.1128/MRA.01156-21

**Published:** 2022-01-27

**Authors:** Nur Arisa Mohd Azim Khan, Sasireigga Jaya Jothi, Erneeza Mohd Hata, Sivachandran Parimannan, Ganesan Vadamalai, Heera Rajandas

**Affiliations:** a Department of Plant Protection, Faculty of Agriculture, Universiti Putra Malaysia, Serdang, Selangor, Malaysia; b Centre of Excellence for Omics-Driven Computational Biodiscovery (COMBio), AIMST University, Bedong, Kedah, Malaysia; c Laboratory of Sustainable Agronomy and Crop Protection, Institute of Plantation Studies, Universiti Putra Malaysia, Serdang, Selangor, Malaysia; University of Arizona

## Abstract

We characterized the draft genome of the potentially beneficial Bacillus tropicus strain UPM-CREST01, which was isolated from the bulk soil at a paddy cultivation area in Kampung Gajah, Perak, Malaysia. The final draft assembly of 5,252,705 bp, with a G+C content of 35.23%, was found to harbor 5,368 coding sequences, including several plant-growth-promoting genes.

## ANNOUNCEMENT

Members of the genus *Bacillus* are thought to possess a variety of positive characteristics that benefit vegetation explicitly or implicitly via nutrient absorption or general growth enhancement via the synthesis of plant growth hormones, including resistance against infections and other abiotic stresses (1). Hence, species of the functionally diverse genus *Bacillus* are among the most utilized microbes throughout the agricultural biotechnology sector (2). Here, we report a draft genome sequence of Bacillus tropicus strain UPM-CREST01, which was isolated from the bulk soil of a high-yielding paddy plot in Kampung Gajah, Perak, Malaysia (4.1841°N, 100.9389°E).

The strain was isolated on nutrient agar following serial dilution of soil and incubation for 24 h at 37°C. The single colony selected was then grown for 24 h at 37°C in Luria-Bertani broth until it reached an optical density of 0.8 at 600 nm (OD_600_) before being subjected to genomic DNA extraction using the NucleoSpin tissue kit (Macherey-Nagel). The concentration of the genomic DNA was determined using an Invitrogen Qubit 4.0 fluorometer, and sequencing libraries were generated using the Nextera XT DNA library preparation kit (Illumina) with 1 ng of input DNA, according to the manufacturer’s guidelines. Upon paired-end sequencing (2 × 150 bp) with the Illumina NovaSeq platform, the reads were subjected to preprocessing using Trimmomatic v0.39 (SLIDINGWINDOW:4:30; MINLEN:80) (3). Trimming resulted in 2,685,616 reads, with a mean length of 150 bases. A *de novo* assembly was then performed using Unicycler v0.4.9 (4) and assessed with QUAST v5.0.2 (5). Default parameters were used for all software, unless stated otherwise.

The assembly resulted in 51 contigs, with an *N*_50_ value of 519,917. The final length of the assembled genome sequence was 5,252,705 bp, with a G+C content of 35.23%. The final genome coverage was 63×. The Similar Genome Finder service available at PATRIC v3.6.12 traced the contigs back to Bacillus tropicus strain SN1, an endophytic isolate with biocontrol ability (6). An average nucleotide identity (ANI) analysis revealed a high level of sequence similarity (99.87%) between the draft genome sequences of the strains.

Using the NCBI Prokaryotic Genome Annotation Pipeline (PGAP) (7), a total of 5,368 coding sequences (CDSs), 70 tRNAs, and 5 noncoding RNAs were identified in the genome of the UPM-CREST01 strain. In addition, the RAST tool kit (RASTtk) (8) successfully identified several key plant-growth-promoting genes in the genome, including genes encoding auxin efflux carrier protein, cytokinin 9-β-glucosyltransferase, and alkaline phosphatase, which boost plant growth and development and improve phosphate solubilization (9–12). The genome also harbors genus-specific siderophore biosynthesis proteins such as bacillibactin and anthrachelin, which are essential for iron acquisition in limiting environmental conditions. Siderophores are said to provide a biocontrol effect due to the competitive advantage they provide the bacteria in chelating Fe efficiently and reducing its availability to pathogenic microbes (13, 14). Therefore, this strain has the potential to be an efficient and nontoxic biocontrol agent that can properly prevent the growth of pathogenic microbes in the ecosystem, while also meeting the growing demand for viable ecology and sustainable agriculture (15). Subsequent *in vitro* analysis of this strain could provide a better understanding of its beneficial roles in paddy growth.

### Data availability.

The whole-genome shotgun project has been deposited in NCBI under the accession number JAJAQE010000000. The associated BioProject, SRA, and BioSample accession numbers are PRJNA763714, SRR15910132, and SAMN21447887, respectively.
